# Genome-wide SNP discovery for development of high-density genetic map and QTL mapping of ascochyta blight resistance in chickpea (*Cicer arietinum* L.)

**DOI:** 10.1007/s00122-019-03322-3

**Published:** 2019-03-16

**Authors:** Amit Deokar, Mandeep Sagi, Bunyamin Tar’an

**Affiliations:** Department of Plant Sciences, College of Agriculture and Bioresources, University of Saskatchewan, Saskatoon, Canada

## Abstract

**Key message:**

A high-density linkage map of chickpea using 3430 SNPs was constructed and used to identify QTLs and candidate genes for ascochyta blight resistance in chickpea.

**Abstract:**

Chickpea cultivation in temperate conditions is highly vulnerable to ascochyta blight infection. Cultivation of resistant cultivars in combination with fungicide application within an informed disease management package is the most effective method to control ascochyta blight in chickpeas. Identifying new sources of resistance is critical for continued improvement in ascochyta blight resistance in chickpea. The objective of this study was to identify genetic loci and candidate genes controlling the resistance to ascochyta blight in recombinant inbred lines derived from crossing cultivars Amit and ICCV 96029. The RILs were genotyped using the genotyping-by-sequencing procedure and Illumina^®^ GoldenGate array. The RILs were evaluated in the field over three site-years and in three independent greenhouse experiments. A genetic map with eight linkage groups was constructed using 3430 SNPs. Eight QTLs for resistance were identified on chromosomes 2, 3, 4, 5 and 6. The QTLs individually explained 7–40% of the phenotypic variations. The QTLs on chromosomes 2 and 6 were associated with the resistance at vegetative stage only. The QTLs on chromosomes 2 and 4 that were previously reported to be conserved across diverse genetic backgrounds and against different isolates of *Ascochyta rabiei* were confirmed in this study. Candidate genes were identified within the QTL regions. Their co-localization with the underlying QTLs was confirmed by genetic mapping. The candidate gene-based SNP markers would lead to more efficient marker-assisted selection for ascochyta blight resistance and would provide a framework for fine mapping and subsequent cloning of the genes associated with the resistance.

**Electronic supplementary material:**

The online version of this article (10.1007/s00122-019-03322-3) contains supplementary material, which is available to authorized users.

## Introduction

Chickpea (*Cicer arietinum* L.) is the world’s second most important grain legume. Multiple pests and diseases significantly affect chickpea productivity. Among the diseases, ascochyta blight caused by the necrotrophic fungal pathogen *Ascochyta rabiei* (Pass.) Lab. is one of the most devastating fungal diseases of chickpea. *A. rabiei* can infect the chickpea plant at any growth stage from plant emergence to seed maturity and produces blight-like symptoms on all above-ground plant parts. The ascochyta blight infection results in lower yield and poor seed quality. Ascochyta blight infection can occur across all major chickpea growing areas globally; however, the frequency of occurrence and severity are much higher in cool and humid growing areas, where several epidemics of ascochyta blight had caused complete crop loss (Kaiser et al. 2000). In North America, management of ascochyta blight is heavily dependent on fungicide applications. This practice has resulted in insensitivity of *A. rabiei* isolates against certain fungicides such as strobilurin (Chang et al. 2007; Wise et al. 2009). To avoid or delay the progress of *A. rabiei* becoming insensitive to fungicides, the use of resistant varieties is considered the most effective and sustainable disease management strategy. Globally, many sources of resistance to ascochyta blight have been identified and were successfully used in chickpea breeding to develop new resistant cultivars (Sharma and Ghosh 2016). However, the levels of resistance tended to decrease due to the rapid adaptation of *A. rabiei* to the resistance. No source of high level of resistance to ascochyta blight has been identified in Canada; however, lines with partial resistance have been identified and were successfully used in breeding programmes to develop new cultivars with improved resistance (Chandirasekaran et al. 2009). Chickpea cultivars with partial resistance to ascochyta blight, such as CDC Frontier and CDC Orion, were developed at the Crop Development Center at the University of Saskatchewan, Canada, using genotypes with partial resistance as the sources of resistance (Taran et al. 2011; Warkentin et al. 2005). The use of partial resistant cultivars in combination with other means of disease management such as selection of field with less or no ascochyta history, use of disease-free and treated seeds, rotation of chickpea every 3–4 years and application of fungicides showed successful ascochyta blight disease control in the Canadian chickpea growing areas.

The partial resistance in the current cultivars contributed to disease management by delaying the onset of the disease, thus affecting the cycles of spore production and spread (Jayakumar et al. 2005). However, the exact genetic and molecular mechanism of partial resistance against *A. rabiei* infection is still unknown. Depending on the isolates of *A. rabiei* and the method of disease scoring, both qualitative and quantitative modes of inheritance for resistance against ascochyta blight have been reported in chickpea. Resistance to ascochyta blight was initially characterized as monogenic with additional modifier genes. Single dominant or recessive gene controlling ascochyta blight resistance in both desi and kabuli types was reported (Dey and Singh 1993; Tewari and Pandey 1986). However, recent studies using recombinant inbred lines demonstrated continuous distribution of disease response and suggested a polygenic inheritance. Multiple QTLs for resistance to ascochyta blight, which individually contributed 12–50% of the total phenotypic variation, have been identified. These QTLs were reported in different mapping populations from inter- and intra-specific crosses and tested at different growth stages (seedling, flowering and pod filling) under greenhouse (controlled) and field conditions using different disease scoring methods (Cho et al. 2004; Collard et al. 2003; Flandez-Galvez et al. 2003; Udupa and Baum 2003). The QTLs on LG2 (QTL_AR3_) and LG4 (QTL_AR1_ and QTL_AR2_) were conserved across different populations (Madrid et al. 2014; Millan et al. 2003; Santra et al. 2000; Stephens et al. 2014; Taran et al. 2007; Tekeoglu et al. 2002; Udupa and Baum 2003). SSR markers linked to these QTLs have been used for marker-assisted backcrossing to introgress the ascochyta blight resistance into adapted chickpea cultivars (Madrid et al. 2012; Taran et al. 2013). Although these QTLs have been successfully used to improve the resistance, the genes underlying these QTLs and the molecular mechanism against ascochyta blight infection are still unknown.

The advances in sequencing technologies have motivated the development of several low-cost sequence-based genotyping methods for genome-wide genetic characterization of large number individuals in genetic mapping and population genetic studies. Among the sequence-based genotyping methods, GBS is a popular choice for SNPs discovery and genotyping to construct high-density linkage maps. GBS has been successfully used for genetic studies in various plants species (Poland and Rife 2012; Sonah et al. 2015), including chickpea (Deokar et al. 2014; Verma et al. 2015; Gaur et al. 2015; Daba et al. 2016). The availability of the sequence-based genetic maps and the high-quality whole-genome sequence of both desi and kabuli chickpeas (Jain et al. 2013; Varshney et al. 2013) would facilitate the identification of the candidate genes and help to understand the mechanism of the resistance against ascochyta blight disease.

The changes in the populations of *A. rabiei* in response to the deployment of resistance cultivars have been observed in chickpea (Mehmood et al. 2017; Vail and Banniza 2008). The study on *A. rabiei* isolates collected from the Canadian Prairies showed a shift in the pathogenicity of the Canadian population of *A. rabiei* over time to higher aggressiveness (Vail and Banniza 2008). This study speculated further changes in the Canadian *A. rabiei* population to even greater aggressiveness as new resistant cultivars are widely grown. Therefore, it is necessary to identify new sources of resistance and to implement them in resistance breeding for the continued development of cultivars with improved resistance. In an effort towards identifying a new source of resistance, cultivar Amit (B90) was identified as resistant to *A. rabiei* isolates from Canada. The present study was conducted to examine the genetic and molecular basis of the resistance in recombinant inbred lines derived from crossing cultivar Amit and ICCV96029 using QTL mapping and candidate gene analysis.

## Materials and methods

### Plant materials

A chickpea mapping population consisting of 133 RILs was developed from a cross between ICCV 96029 and Amit. ICCV 96029 is a desi-type genotype and is highly susceptible to ascochyta blight, whereas Amit (also known as B90) is a partially resistant kabuli cultivar selected from within a Bulgarian landrace (Anbessa et al. 2009; Taran et al. 2007). The individual F_2_ plants were advanced to F_8_ by the single-seed descent method. The ICCV 96029 × Amit RIL population hereafter is referred to as CPR-02.

The CPR-02 RILs along with the parental genotypes ICCV 96029 and Amit were screened for ascochyta blight response under controlled (greenhouse) and under field conditions at Elrose (51.2006°N, 108.0329°W), Saskatchewan (SK), Canada, in 2014 and 2015 and at Saskatoon (52.1332°N, 106.6700°W), SK, Canada in 2017.

In the greenhouse experiments, plants were grown in 0.5-L pots filled with Sunshine^®^ mix#4 growth medium (Sun Gro Horticulture Canada Ltd). All the greenhouse experiments were conducted with three replications, and pots were arranged in a completely randomized design. The day/night temperature of 20/16 °C and photoperiod of 16 h were maintained throughout the experiment using artificial light sources. To evaluate the plant response against ascochyta blight at the vegetative growth stage, 3-week-old seedlings at about 8–10 internode stage were inoculated with *A. rabiei* isolate AR170-3 as described in Taran et al. (2007) and Anbessa et al. (2009). Conidial concentrations of 2 × 10^5^ pycnidiospores mL^−1^ were used and 3 mL conidial suspension per plant was sprayed. The AR-170-3 is a moderately aggressive isolate of *A. rabiei* collected from chickpea fields in Saskatchewan, Canada, and is pathogenic to all the tested genotypes at the CDC chickpea breeding programme. The disease response was evaluated 3 weeks after inoculation at pre-flowering stage using a 0–9 rating scale (Singh and Reddy 1993; Chongo et al. 2004, Taran et al. 2007). The 0–9 rating scale is as follows: 0 = no symptoms, 1 = few, very small (< 2 mm^2^) lesions on leaves and/or stems, < 2% plant area affected (PAA); 2 = very small (< 2 mm^2^) lesions, 2–5% PAA; 3 = many small lesions (2–5 mm^2^), 5–10% PAA; 4 = many small lesions, few large (> 5 mm^2^) lesions, 10–25% PAA; 5 = many large lesions, 25–50% PAA; 6 = lesions coalescing, 50–75% PAA, 7 = lesions coalescing with stem girdling, 75–90% PAA; 8 = stem girdling or breakage, > 90% PAA; 9 = plants dead. The entire disease screening in the greenhouse was repeated three times following the same protocol.

In each year, each RIL was planted in three rows 1 m × 1 m microplot with three replications and arranged as a randomized complete block design. No artificial inoculation was provided. Disease rating was done on the plot basis during the pod-filling stage (R5) using the 0–9 scale as described above. At the Saskatoon location in 2017, each RIL was planted in a 3.8-L pot filled with Sunshine^®^ mix#4 growth medium (Sun Gro Horticulture Canada Ltd). The RILs were planted in three replications and arranged as randomized complete block design in the yard of the Crop Science Field Laboratory of the Department of Plant Sciences, University of Saskatchewan. Similarly, at Saskatoon, the plants were exposed to natural infection of ascochyta blight throughout the growing cycle. Disease rating was done at the pod-filling stage (R5) using the same rating scale as stated above.

### Phenotypic data analysis

Statistical analysis was conducted using SAS software (version 9.4, SAS Institute Inc., Cary, North Carolina, USA). In the greenhouse experiment, individual and combined analyses across the three experimental repeats were completed. Before performing the analysis of variance (ANOVA), the homogeneity of variance and the normality of residuals were tested using Levene’s and Shapiro–Wilk normality tests, respectively. ANOVA was done using PROC MIXED, in which the genotypes were considered as a fixed factor and repeats were considered as a random factor. The LSMEANS statement was used to compute the average ascochyta blight score for each RIL.

For field evaluation, both combined analyses across locations and years and separate analysis on each year (2014, 2015 and 2017) were conducted. ANOVA was done using PROC MIXED in which genotypes were considered as a fixed factor and years as a random factor. The LSMEANS statement was used to compute the average ascochyta blight score for each RIL. For a separate analysis of each year, ANOVA was done using the PROC MIXED procedure, in which the RILs were considered as a fixed factor and replication was considered as a random factor. To estimate the broad sense heritability (*H*^2^), variance components were calculated using the SAS PROC VARCOMP procedure (SAS Institute 1999). The *H*^2^ of ascochyta blight response at plot level based on individual experiment and over the years was estimated using the following two equations, respectively:

$$ H^{2} = \frac{{\sigma^{2} G}}{{\sigma^{2} G + \sigma^{2} {\text{er}}}} \,{\text{and}}\, H^{2} = \frac{{\sigma^{2} G}}{{\sigma^{2} G + \sigma^{2} GY + \sigma^{2} {\text{er}}}} $$

where *σ*^2^G, *σ*^2^Y, *σ*^2^GY and *σ*^2^er are estimates of genotype, site-year, genotype by site-year interaction and error variance, respectively (Singh et al. 1993). Pearson’s correlation coefficients were calculated between field and greenhouse disease data.

### SNP genotyping

High-molecular weight genomic DNA from young leaf tissue of a single plant of ICCV 96029, Amit and CPR-02 RILs (F_8_) was isolated using the modified CTAB protocol. *Ribonuclease* A (*RNase* A)-treated genomic DNA was quantified using NanoDrop™ 8000 spectrophotometer (Thermo Scientific, http://www.thermoscientific.com) and normalized as per requirement of the genotyping platform.

The CPR-02 RILs were genotyped using the single-enzyme (*ApeKI*)-based genotyping-by-sequencing approach (Elshire et al. 2011). Library construction and paired-end (PE) sequencing using Illumina^®^ HiSeq 2000 were done at the BGI@UC Davis, next-generation sequencing facility. The GBS reads were processed using GBS pipeline developed at the Pulse Bioinformatics Core facility at the Department of Plant Sciences, University of Saskatchewan, Canada (http://carolyncaron.github.io/GBSpipeline/). The GBS pipeline is a Perl script which launches demultiplexing, trimmomatic (trimming and filtering), bowtie2 (alignment to a reference genome) and SAMtools and BCFtools (SNP calling). Raw SNPs were further filtered using VCFtools to retain SNPs with less than 35% missing data and a minor allele frequency greater than 0.3.

We have previously developed Illumina^®^ GoldenGate SNP array of 1536 genic SNPs (Deokar et al. 2014). The CPR-02 RILs were also genotyped using the chickpea Illumina^®^ GoldenGate 1536-SNP array as per manufacturer’s protocol and as described in Deokar et al. (2014). Briefly, the chickpea GoldenGate 1536-SNP array raw hybridization intensity data were processed for allele calling using GenTrain clustering algorithm implemented in GenomeStudio software version 2010.3 (Illumina Inc., San Diego, CA). We selected SNP markers with the GenTrain score ≥ 0.7 and missing rate < 20%. The GenTrain score for a SNP reflects the accuracy of SNP clustering. The higher the GenTrain scores, the more reliable the SNP genotypic data.

### Map construction

The SNP data from both genotyping platforms were used to construct the CPR-02 linkage map. Genotype scores were converted into ABH format as A: ICCV 96029 genotype, B: Amit genotype, H: heterozygote, and − 1 as missing genotype. Redundant SNP markers with a correlation coefficient of 1.0 were classified into one bin, and a representative marker with the lowest missing data was selected to represent that bin. The bin grouping of the redundant SNP markers was performed using BIN functionality employed in QTL ICIMapping 3.2 software (Meng et al. 2015). A single marker representing each bin was used for linkage map construction using the same software (Meng et al. 2015). First, all markers were grouped into eight linkage groups with the LOD threshold of 5.0. Markers within a linkage group were ordered using recombination counting and ordering algorithm (RECORD). The final marker order was fine-tuned by rippling with a number of recombination events (COUNT) algorithm with a window of five markers. Map distance in centimorgan (cM) was calculated using the Kosambi mapping function. The final recombination bin map was visualized using MapChart 2.2 software (Voorrips 2002).

### QTL mapping

QTL mapping was done using inclusive composite interval mapping of additive and dominant QTL (ICIM-ADD) module of QTL ICIMapping 3.2 software. A genome-wide LOD significant threshold was calculated for each set of phenotypic data using 1000 permutation test at a significant level of *P* < 0.05.

### Positioning QTL on the physical map

In order to physically map the previously reported ascochyta blight QTLs on the chickpea genome, the primer sequences and the expected amplicon size of the QTL flanking microsatellite markers were extracted from the related publications. Both forward and reverse primer sequences were used for NCBI’s BLASTN against the chickpea reference genome. The BLASTN results of the top three perfect matches were extracted and analysed for the base-pair locations of the beginning of the forward primer and the end of the reverse primer. The location of the SSR marker on the physical map was confirmed if both forward and reverse primer sequences are found within the expected amplicon size. The QTL location on the physical map was confirmed if both QTL flanking markers were mapped to the identical chromosome.

### Candidate gene analysis and marker development

Based on the physical position of the QTLs on the chickpea genome, the gene sequences were retrieved. Candidate genes were selected based on the gene annotation and prior information of their involvement in disease resistance. The SNPs between Amit and ICCV 96029 that are located within the candidate gene region were selected and used to design KASP (Kompetitive Allele-Specific PCR) markers. The KASP assay was carried out in a StepOnePlus™ Real-Time PCR System (Applied Biosystems), and the end-product fluorescence readings were analysed using StepOne Software v2.1.

### Genetic mapping of candidate genes in CPR-02

The candidate genes that were physically located within the QTL intervals based on the CDC Frontier reference genome were genetically mapped in the CPR-02 together with the SNP data from the GBS and GoldenGate assay. The map was constructed using ICIMapping 3.2 software as described above. This linkage map was further used in QTL analysis to validate the position of the candidate genes and the QTLs.

## Results

### SNP genotyping using the genotyping-by-sequencing approach and Illumina^®^ GoldenGate array

Paired-end sequencing of the RILs and the two parents (totalling 135 individuals) generated over 224.4 million reads with an average of 1.7 million reads per line. On average, 74.6% (ranged from 70.2 to 79.7%) of the reads were aligned to the CDC Frontier reference genome sequence (v2.6) and the remaining 25.4% (ranged from 20.3 to 29.8%) reads were removed from further analysis. A total of 15,581 SNPs were identified between ICCV 96029 and Amit. Among them, 14,531 SNPs were physically mapped on the eight chickpea chromosomes, whereas the remaining 1050 SNPs were mapped on 188 unplaced scaffolds. Chromosome 4 had the highest proportion of the mapped SNPs (19.7%; 3065 SNPs), whereas chromosome 8 had the lowest proportion of the mapped SNPs (3.0%; 474 SNPs).

The Illumina GoldenGate assay generated a total of 1522 SNPs (99.1%) that exhibited clear clustering patterns and high GenTrain scores (mean = 0.84 ± 0.10 s.d.). Of these, 664 (43.3%) SNPs were polymorphic between ICCV 96029 and Amit, and the remaining 841 (54.8%) were monomorphic. Seventeen (1.1%) SNPs failed to generate genotype score and 14 (0.9%) had a pattern which both parents failed to generate SNP score.

### Construction of CPR-02 linkage map

A total of 3492 SNPs including 2828 SNPs from the GBS and 664 SNPs from the Illumina^®^ GoldenGate™ array were combined to generate a high-density linkage map. The majority of the markers (3430 SNPs) were grouped into eight linkage groups (LG) that equalled to the eight haploid chickpea chromosomes, whereas the rest 62 SNPs remained unlinked to any of the linkage groups. The map covered a total length of 867.9 cM with an average distance between the neighbouring SNP loci of 0.2 cM. LG 4 contained the largest number of SNPs (1430 SNPs), whereas LG5 contained the smallest number of 37 SNPs. The largest linkage group LG7 spanned 128.2 cM, whereas the smallest linkage group LG8 spanned 59.6 cM (Table 1).

**Table 1:** Summary statistics of the chickpea CPR-02 linkage map

Linkage groups	Number of SNP markers	Number of independent marker loci^a^	Number of BIN markers	No of singleton markers	Map distance (cM)	Average distance between two markers	Number of gap ≥ 10 cM
Ca1	402	54	27	27	114.4	2.1	1
Ca2	405	47	28	19	97.0	2.0	2
Ca3	338	59	23	36	83.9	1.4	0
Ca4	1430	83	51	32	122.7	1.5	0
Ca5	37	32	2	30	112.1	3.0	3
Ca6	366	67	30	37	117.2	1.7	0
Ca7	340	64	23	41	128.2	2.0	0
Ca8	112	23	10	13	59.6	2.6	1
Total	3430	429	194	235	835.0	1.9	7

^a^Number of independent marker loci includes the number of BIN markers and the number of singletons

The 3430 mapped SNPs were grouped into 194 recombination bins and 235 single markers. From the bin markers, individual SNP markers representing the corresponding recombination bin with the lowest missing values were selected for bin-based linkage map construction. A minimum of two to a maximum of 361 SNP markers were grouped into different recombination bins. LG4 constituted the highest (51) recombination bins and 32 single markers, whereas LG5 constituted only two recombination bins and 30 single markers (Table 1).

### The response of CPR-02 RILs to *A. rabiei* infection under field and greenhouse conditions

The visible symptoms of ascochyta blight on the leaves began to appear at 7–8 days after inoculation and were scored at 21 days after inoculations, at which time the plants were at the pre-flowering stage. Under the greenhouse experiments, the ascochyta blight score of the susceptible parent ICCV 96029 ranged from 6.8 to 7.8, and the ascochyta blight score of the resistance parent Amit ranged from 3.5 to 4.5. The mean ascochyta blight score of the CPR-02 population was 5.0 ranging from 3.0 to 8.0. The coefficient of variation (CV) of the ascochyta blight disease score under greenhouse screenings ranged from 14.7 to 20.8% (Supplementary Table 1). The ANOVA showed that the CPR-02 RILs had significant variation (*P* < 0.001) of the ascochyta blight severity (Table 2). The interaction of genotype by repeat was not significant and, therefore, data from all three greenhouse repeated experiments were combined and the mean estimates were used for QTL analysis (Table 2).

**Table 2:** Analysis of variance for the ascochyta blight scores of the CPR-02 population under greenhouse conditions (three repeated experiments) and field conditions at Elrose, SK, in 2014 and 2015 and at Saskatoon, SK, in 2017

Year/locations	Effect	*F* value
Greenhouse (three repeats)	*G*	7.14***
	*R*	0.48^ns^
	G*R	0.36^ns^
	*σ* ^2^ *G*	0.77
	*σ* ^2^ *GR*	0.00
	*σ*^2^er	0.52
	*H* ^2^	0.59
Field combined years (2014, 2015, 2017)	*G*	8.84***
	*Y*	230.67***
	G*Y	3.27***
	*σ* ^2^ *G*	0.34
	*σ* ^2^ *Y*	0.32
	*σ*^2^G*Y	0.46
	*σ*^2^er	0.43
	*H* ^2^	0.28
Individual year/location		
Elrose 2014	*G*	5.05***
	*σ* ^2^ *G*	0.58
	*σ*^2^er	0.33
	*H* ^2^	0.65
Elrose 2015	*G*	6.53***
	*σ* ^2^ *G*	0.49
	*σ*^2^er	0.53
	*H* ^2^	0.48
Saskatoon 2017	*G*	8.48***
	*σ* ^2^ *G*	1.19
	*σ*^2^er	0.48
	*H* ^2^	0.71

*G* genotypes, individual RILs from CPR02 population, *R* experimental repeats in the greenhouse, *Y* year, G*R genotype by repeat interaction, G*Y genotype by year interaction. *σ*^2^*G*, *σ*^2^*GR*, *σ*^2^*GY* and *σ*^2^*er* estimate of genotypic, genotype by repeat, genotype by year and error variance, respectively. *H*^2^ is broad sense heritability

*** indicates a significant difference at *P* ≤ 0.001 and ns = non-significant

Three field disease screenings were conducted in 2014, 2015 and 2017. Under field screening, the disease rating was done at the pod-filling stage. The ascochyta blight score for the resistant parent Amit ranged from 4.0 to 4.5, whereas the score of the susceptible parent ICCV 96029 ranged from 7.5 to 8.0 under field conditions. Significant differences were detected among the RILs scores in response to ascochyta blight infection. The mean ascochyta blight score of the CPR-02 RILs was 5.7 ranging from 3.0 to 9.0. Environmental conditions played an important role in disease development and progression. The differences in annual weather conditions may have affected disease development under field conditions; therefore, both combined and individual year analyses of variance (ANOVA) were conducted (Table 2). The results showed that the genotype (the CPR-02 RILs) had a significant effect at *P* < 0.001 on ascochyta blight severity in both combined and individual year analyses (Table 2). There was a significant effect of environment (year) and genotype by environment (year) interaction on CPR-02 ascochyta blight scores; therefore, disease scores from individual years were used in separate QTL analyses. Correlation analysis showed a significant positive correlation between the greenhouse and field disease response of the CPR-02 RILs against ascochyta blight infection (Supplementary Table 2).

### Mapping QTLs for ascochyta blight resistance

**Fig. 1:**
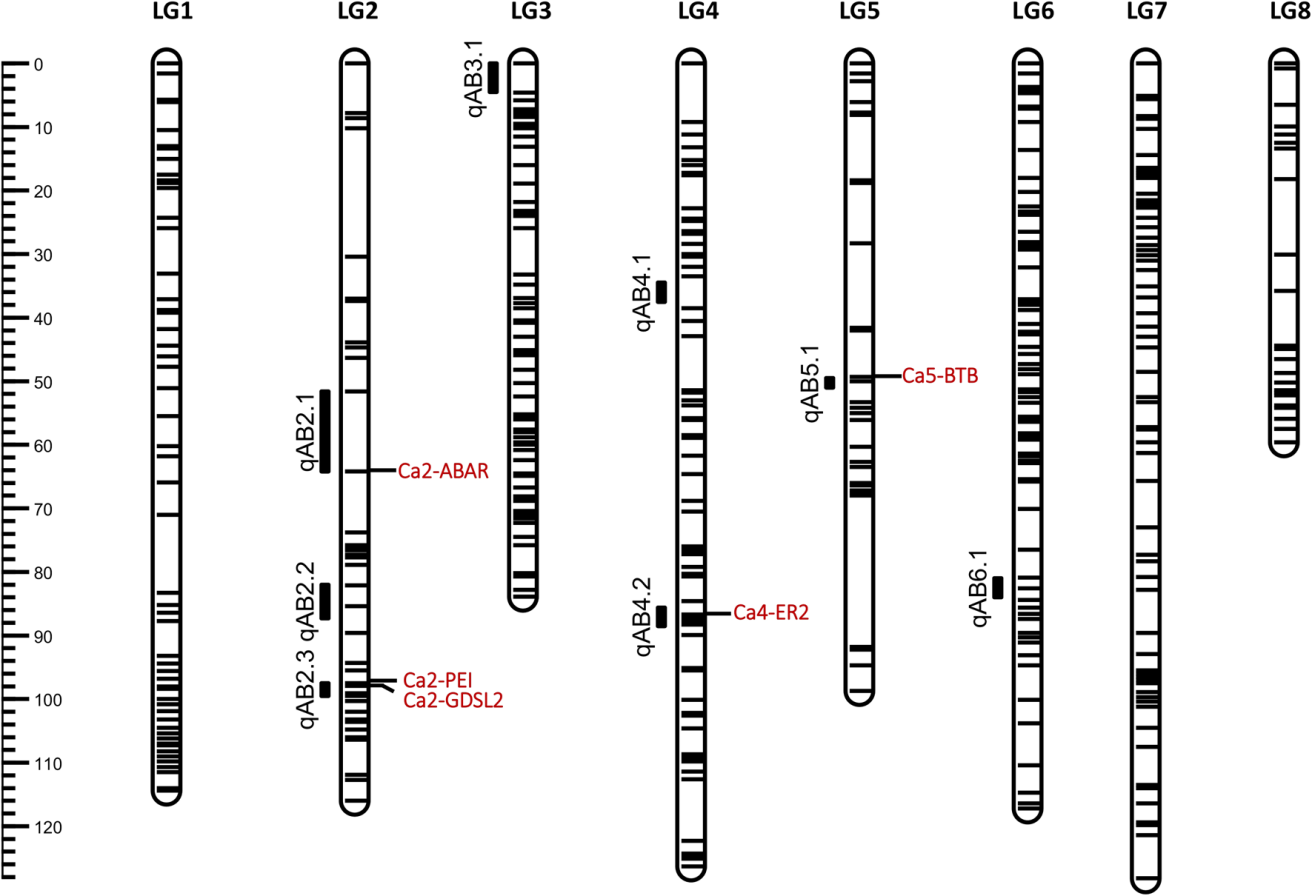
Linkage map of chickpea RILs with eight linkage groups (LG1–LG8) showing the quantitative trait loci (QTLs) for resistance to ascochyta blight under field and greenhouse conditions and the candidate genes. The scale on the left side indicates the genetic distance in centimorgan (cM). Eight QTLs for ascochyta blight resistance are shown on the left side of the corresponding linkage group. Candidate genes co-localized with the QTLs are depicted in red colour on the right side of corresponding linkage group

Eight QTLs associated with the resistance to ascochyta blight were identified on five out of eight chickpea chromosomes (Fig. 1 and Table 3). The amount of phenotypic variation explained by individual QTLs ranged from 6.6% (qAB2.2) to 40% (qAB5.1). Two QTLs on chromosome 2 (qAB2.1 and qAB2.3) and one on chromosome 6 (qAB6.1) were identified specifically from the disease screening conducted in the greenhouse. The qAB3.1 was detected consistently in both field (Elrose 2014) and greenhouse screenings. The qAB4.1, qAB4.2 and qAB5.1 were consistently detected in 2-year disease screenings under field conditions. The QTLs particularly detected in the greenhouse screenings could be specifically associated with vegetative stage resistance as the RILs were inoculated and evaluated for disease response during the vegetative growth stage just before flowering, whereas those QTLs specifically detected under field conditions could be considered as regenerative stage-specific QTLs as the disease response was evaluated during the seed filling period. The resistant parent (Amit) contributed the alleles for the resistance at all QTLs that were detected in this study.

**Table 3:** List of quantitative trait loci (QTL) associated with the resistance to ascochyta blight in a recombinant inbred line population derived from a cross between ICCV 96029 (susceptible) and Amit (resistant) chickpea cultivars

QTL	Environment	Chromosome	Position (cM)	Marker interval	LOD	Add	PVE (%)
		Ca2					
qAB2.1	GH		56.0	Ca2-ABA-R- Cav1sc520.1p50440	4.5	0.3	13.8
qAB2.2	EL2015		83.5	Cav1sc246.1p121732-Cav1sc689.1p195825	4.2	0.3	6.6
qAB2.3	GH		99.0	Ca2-GDSL2- Ca2-PEI	3.2	0.3	11.8
		Ca3					
qAB3.1	GH		1.0	SCA3_15444471- SCA3_21346384	3.1	0.3	9.4
	EL2014		1.0	SCA3_15444471- SCA3_21346384	4.0	0.2	6.7
		Ca4					
qAB4.1	EL2015		34.5	SCA4_6022188- SCA4_8584363	6.5	0.3	11.2
	SAS2017		34.5	SCA4_6022188- SCA4_8584363	3.2	0.4	9.4
qAB4.2	EL2014		87.0	SCA4_21540251- Ca4-ER2	6.8	0.4	20.6
	El2015		87.0	SCA4_21540251- Ca4-ER2	6.1	0.4	16.5
		Ca5					
qAB5.1	EL2014		49.5	SCA5_20906121 Ca5-BTB	3.9	0.3	11.0
	EL2015		49.5	SCA5_20906121 Ca5-BTB	19.0	0.6	40.2
		Ca6					
qAB6.1	GH		82.0	SCA6_54348174-Cav1sc30.1p60427	4.1	0.4	15.2

### Genomic regions and gene-based SNP markers associated with resistance to ascochyta blight

The sequences of the regions flanking the SNPs were used to anchor the QTLs to the chickpea physical map. The QTL intervals for qAB2.1, qAB2.2 and qAB2.3 on chromosome 2 corresponded to a physical distance of around 3.0, 0.9 and 1.6 Mb on the pseudochromosome Ca2 of the CDC Frontier genome assembly V2.6 (Supplementary Table 3). The QTL qAB3.1 interval on chromosome 3 corresponded to a physical distance of around 6 Mb on pseudochromosome Ca3. The QTL intervals for qAB4.1 and qAB4.2 on chromosome 4 corresponded to a physical distance of around 2.5 and 5.2 Mb on pseudochromosome Ca4. The qAB5.1 interval on chromosome 5 corresponded to a physical distance of around 0.17 Mb on pseudochromosome Ca5 and was the smallest QTL interval in terms of physical distance. The qAB6.1 interval corresponded to a physical distance of around 10.4 Mb on pseudochromosome Ca6 and was the largest QTL interval in terms of physical distance. Following the annotation, several genes previously identified as candidate genes located within the physical interval of the QTL and involved in disease resistance were selected. The sequences of these genes in ICCV 96029 and Amit were retrieved from the in-house whole-genome sequence database. Sequence polymorphisms between ICCV96029 and Amit were detected in the GDSL esterase/lipase (GDSL), PECTIN ESTERASE INHIBITOR (PEI), ABA receptor (ABA-R), ethylene receptor (ER) and BTB/POZ domain protein (BTB) genes. The sequence information was used to generate KASP assays for SNP genotyping (Table 4, Supplementary Table 4). The CPR-02 RILs were genotyped using these SNP markers, and a new genetic map was constructed combining the SNPs from the GBS and GoldenGate array and the KASP markers for the candidate genes. The KASP marker (Ca2-ABA-R) for ABA-R was genetically mapped on LG2 and overlapped with the qAB2.1, whereas the candidate gene GDSL (KASP marker: Ca2-GDLS2) and PEI (KASP marker: Ca2-PEI) were overlapped with qAB2.3 on the same linkage group. The ER2 gene marker was genetically mapped on the qAB4.2, and BTB gene was genetically mapped on qAB5.1 on chromosomes 4 and 5.

**Table 4:** The primer sequences of the KASP SNP markers for the candidate genes associated with the resistance to ascochyta blight with their physical and genetic position on the genome

Candidate gene	KASP Primers	Primer sequences (5′-3′)
ABA receptor (ABA-R), SNP locations: Ca2: 17335866 bp Genetic location: LG2, 64.18 cM	Ca2-ABAR_A1	GAAGGTGACCAAGTTCATGCTTCTTCTTTCATTTGGAATGATGATAACTG
	Ca2-ABAR_A2	GAAGGTCGGAGTCAACGGATTTTCTTCTTTCATTTGGAATGATGATAACTA
	Ca2-ABAR_C1	AATAAATTAACACTAGCAAGTAGCAACCAT
Pectin esterase inhibitor (PEI), SNP locations: Ca2: 20675626 bp Genetic location:LG2, 97.9 cM	Ca2-PEI-A	GAAGGTGACCAAGTTCATGCTAACAAACACTGAAAACTCGTACTTCGT
	Ca2-PEI-B	GAAGGTCGGAGTCAACGGATTCAAACACTGAAAACTCGTACTTCGC
	Ca2-PEI-C	GGCACCAGGTCCAATATTTCCAAACT
GDSL esterase/lipase. SNP locations: Ca2: 20836065 bp Genetic location: LG2, 99.12 cM	Ca2-GDSL2-A	GAAGGTGACCAAGTTCATGCTGTATTGATTTTCTTAACCACAAACCAACA
	Ca2-GDSL2-B	GAAGGTCGGAGTCAACGGATTGTATTGATTTTCTTAACCACAAACCAACT
	Ca2-GDSL2-C	CCAATAAAATCAGCAGCATTCTTTCCGTT
Ethylene Receptor (ER2), SNP locations: Ca4: 26669758 bp Genetic location: LG4, 87.5 cM	Ca4-ER2_A1	GAAGGTGACCAAGTTCATGCTCCACCTCAACTATCTCCAACTCC
	Ca4-ER2_A2	GAAGGTCGGAGTCAACGGATTAACCACCTCAACTATCTCCAACTCT
	Ca4-ER2_C1	TTGGTAATGCATAGTGGAGATGTGAGAAT
BTB/POZ domain protein (BTB), SNP locations: Ca5: 21049844 bp Genetic location: LG5, 50.03 cM	Ca5-BTB/POZ_A1	GAAGGTGACCAAGTTCATGCTAAGTCTAAAGAGTTGCCACATCCG
	Ca5-BTB/POZ_A2	GAAGGTCGGAGTCAACGGATTGAAGTCTAAAGAGTTGCCACATCCT
	Ca5-BTB/POZ_C1	AGAGGAATCTAAAGGGAGGGTGCTT

*A1* SNP allele 1-specific primer, *A2* SNP allele 2-specific primer and *C1* common primer, location of candidate gene and SNPs are based on the CDC Frontier reference genome assembly v2.6

## Discussion

### SNP discovery using GBS and linkage map development

Discovery of SNPs and other genetic variants is a key step in several genetic studies including linkage mapping, QTL analysis, population genetic analysis and association mapping. Among the different genetic variants, SNPs are predominantly used as molecular markers in genetic studies due to their abundance in the genome. The quality and the large number of genome-wide markers have a major impact on the genetic analysis as a greater number of markers provides higher resolution genetic map (Xu et al. 2017), higher statistical power in GWAS analysis (Spencer et al. 2009) and increased prediction accuracy in genomic selection (Ma et al. 2016).

The reduced representation sequencing-based genotyping methods including restriction-site-associated DNA (RAD) sequencing (RAD-seq) and GBS have been demonstrated to be an efficient method of high-throughput SNP discovery and genotyping. The reduced representation techniques ensure re-targeting consistent portions of the genome for sequencing across multiple samples and allow low cost per sample and per marker data point (Elshire et al. 2011; Scheben et al. 2017). With the availability of a large number of genome-wide SNPs and advances in microarray technology, a variety of SNP array platforms have been developed which mainly includes Affymetrix Axiom and Illumina’s GoldenGate arrays. Multiple SNPs arrays covering from a few 100s to 60,000 SNPs were developed in chickpea and used to analyse genetic diversity, marker–trait association studies, linkage mapping and QTL analysis (Daba et al. 2016; Deokar et al. 2014; Diapari et al. 2014; Roorkiwal et al. 2018). Overall, the availability of these high-throughput SNP genotyping tools has greatly increased the scale of marker application in genetic mapping and marker–trait association in chickpea.

The NGS-based GBS method and the GoldenGate genotyping array used in the current study allowed the development of a linkage map consisting of 3430 markers (429 recombination bins) covering a total map distance of 868 cM and average marker density of 0.2 cM. These results are comparable to the marker density of chickpea genetic maps constructed using the GBS approach (Verma et al. 2015; Gaur et al. 2015). Overall, the results indicated that the CPR-02 has a sufficient number of markers to capture the majority of the recombination events in the population resulting in increased precision in QTL mapping and candidate gene identification.

### QTL mapping for ascochyta blight resistance

Genomic regions on chromosomes 2 and 4 have consistently been reported to contain QTLs for ascochyta blight resistance in multiple resistant sources using different isolates of *A. rabiei.* The results indicated that these are conserved QTLs associated with ascochyta blight resistance (Sharma and Ghosh 2016). The present study also identified QTLs on chromosomes 2 and 4 (Table 3). Physical mapping of the previously reported QTLs for resistance to ascochyta blight using the flanking microsatellite marker sequences revealed that the QTLs near the markers TA37-GA16 located at 4.7–20.7 Mb on chromosome 2 (Cho et al. 2004), *QTL1* flanked by markers TR19-TA110 physically located at 13.6–35.6 Mb (Anbessa et al. 2009), qAB1.1 flanked by SNP markers scaffold58p286681–scaffold905p693958 physically located at 15.2–17.0 Mb (Daba et al. 2016), candidate gene *Ein3* located at 18.9 Mb (Madrid et al. 2014) and the QTLs identified in the present study qAB2.1 (14.3–17.4 Mb), qAB2.2 (18.3–19.3 Mb) and qAB2.3 (20.7–20.9 Mb) were all located on the same genomic region on chromosome 2 and are referred to as QTL_AR3_ (Supplementary Table 5). Recently, using QTL sequencing approach we identified the same genomic region on chromosome 2 (18.2–18.9 Mb) that was associated with ascochyta blight resistance (Deokar et al. 2018). Two candidate genes GDSL2 (GDSL esterase/lipase) and PEI (pectin esterase inhibitor) were also genetically mapped to the same genomic region. The Arabidopsis GDSL LIPASE-LIKE 1 gene regulates the systemic resistance against the necrotrophic fungus *Alternaria brassicicola* through the ethylene signalling pathway (Kwon et al. 2009). Similarly, overexpression of Arabidopsis PMEI genes showed to increase the pectin methyl esterification of the pectin cell wall and reduce plant susceptibility to fungal and bacterial necrotrophs (Raiola et al. 2010). Considering the role of these two genes in plant defence against the necrotrophic pathogens and their co-localization with the conserved and stable QTL for ascochyta blight resistance, the GDSL and PEI can be the candidate genes. The sequence variations within these genes can be used as molecular markers for marker-assisted selection.

On chromosome 4, two major clusters of QTLs were found on 4–8 Mb (referred as QTL_AR1_) and 22–43 Mb (referred as QTL_AR2_) genomic regions. In the QTL_AR1_ region, qAB4.1 (6.0–8.6 Mb) identified in the present study overlapped with the previously reported QTL_AR1_ flanked by NCPGR91-GAA47 located at 4.4–8.0 Mb (Madrid et al. 2013), qAB4.1 flanked by SNP markers scaffold1758p1006151–scaffold405p3450200 (6.9–13.4 Mb) (Daba et al. 2016) and a QTL flanked GA24-GAA47 (8.0–8.8 Mb) (Cho et al. 2004). The ethylene receptor-like sequence (CaETR-1) was located at 4.3 Mb within the QTL_AR1_ interval and explained up to 33.8% of the total phenotypic variation for ascochyta blight resistance (Madrid et al. 2013). This QTL region has been narrowed down to 4.3–4.5 Mb genomic region using NGS-based BSA sequencing. A candidate gene Ca-AKL18 (AT‐HOOK MOTIF CONTAINING NUCLEAR LOCALIZED 18) gene was identified within this region (Kumar et al. 2018). The Ca-AKL18 showed structural variation in the promoter region and had higher expression under ascochyta blight infection in the moderately resistant parent FLIP84‐92C, suggesting that this gene is the most probable candidate involved in resistance to ascochyta blight in chickpea (Kumar et al. 2018). Although the QTL interval of qAB4.1 overlapped with the QTL interval of QTL_AR1_, fine mapping of this region by Kumar et al. (2018) and Madrid et al. (2012) suggested that the qAB4.1 identified in cultivar Amit (genomic region 6.0–8.6 Mb) could be different than the fine-mapped QTL region of FLIP84-92C.

Another QTL on chromosome 4 identified in the present study (qAB4.2) located at 21.5–26.7 Mb genomic region overlapped with QTL_AR2_ flanked by TA146-TA72 physically located at 24.4–43.6 Mb (Iruela et al. 2006), QTL2 flanked by TA146-TA2 physically located at 24.4–41.9 Mb (Taran et al. 2007), ab_QTL1 flanked by TA146-TA72 markers physically located at 24.4–43.6 Mb (Stephens et al. 2014), AB-Q-SR-4-2 flanked by CaM2049-H4G11 markers and physically located at 31.8–41.7 Mb (Sabbavarapu et al. 2013), qABR4.2 located at 26.2–31.77 Mb (Kumar et al. 2018) and qAB4.1, qAB4.2 and qAB4.3 (24.4–31.0 Mb) (Deokar et al. 2018). An ethylene receptor (ER2) a member of the ethylene signalling pathway was found overlapping (26.6 Mb) with the qAB4.2 identified in the present study. Additionally, several other genes with potential involvement in disease responses such as vegetative cell wall protein, aspartic protease, leucine-rich repeat extensins (LRXs) and protein casein kinase (CK) were also identified in this QTL interval. The common QTLs across multiple resistant genotypes on chromosomes 2 and 4 suggested that there are potentially common genes and mechanism that regulate resistance to ascochyta blight in chickpea. However, to precisely identify and characterize the genes in these regions may require fine mapping with large F_2_ population.

Apart from the common QTLs on chromosomes 2 and 4, three QTLs, one each on chromosomes 3, 5 and 6, were also identified in the present study. The qAB3.1 in the present study overlapped with QTL2 identified earlier in the resistant cultivars CDC Frontier and Amit near SSR marker TA64 (Anbessa et al. 2009; Taran et al. 2007). The qAB3.1 and QTL2 regions are specific to CDC Frontier and Amit. The QTL on chromosome 5 (qAB5.1) is a unique QTL identified in the present study. The QTL qAB5.1 was identified under field screenings and explained up to 40.2% phenotypic variation for response to ascochyta blight disease. The QTL on chromosome 6 was mapped to a large genomic region of 28.4–43.9 Mb. This region was only identified under greenhouse screenings and possibly associated with the resistance to ascochyta blight during the vegetative stage. Differential response of ascochyta blight resistance at seedling (vegetative stage) and adult plant (reproductive stage) was previously observed in chickpea (Collard et al. 2003; Kottapalli et al. 2009).

This study provided insight into the quantitative resistance to ascochyta blight in chickpea. A high-density sequence-based genetic map of chickpea facilitated anchoring the QTLs to physical map and provided a means to align the previously reported QTLs with the QTLs identified in the present study. The high-resolution genetic map of CPR-02 also enabled identification of QTLs in a narrow physical interval as compared to the previously identified QTLs with SSR markers. This study provides tightly linked and robust SNP markers in a form of KASP assay for marker-assisted selection of ascochyta blight resistance in chickpea. The KASP-based SNP markers enabled for automation and high-throughput genotyping and breeder-friendly assay.

#### Author contribution statement

AD and MS collected and analysed phenotypic data. AD generated SNP genotyping data and conducted linkage maps and performed QTL analysis. AD, MS and BT wrote the manuscript. BT conceived the project and guided the analyses. All authors read and approved the final manuscript.

## Electronic supplementary material

Below is the link to the electronic supplementary material.

Supplementary material 1 (DOCX 26 kb)
