# Supplementary material for: Genome-wide SNP discovery for development of high-density genetic map and QTL mapping of ascochyta blight resistance in chickpea (Cicer arietinum L.)
Source: Theor Appl Genet. 2019 Mar 16;132(6):1861–72. doi: 10.1007/s00122-019-03322-3 (PMC6531409; doi:10.1007/s00122-019-03322-3)
Supplement: Supplementary file 1 — Supplementary material 1 (DOCX 26 kb) [file 122_2019_3322_MOESM1_ESM.docx]

|  | | **ICCV 90269** | **Amit** | **CPR02** | | |
| --- | --- | --- | --- | --- | --- | --- |
| **Environment/Location** | **Repeat/year** | **Mean**  **± SD** | **Mean**  **± SD** | **Mean ± SD** | **Range** | **C.V (%)** |
| Greenhouse | Repeat 1 | 7.0±0.8 | 4.5±0.5 | 5.1 ± 1.3 | 3.0-8.0 | 20.8 |
|  | Repeat 2 | 7.8±1.2 | 4.2±0.8 | 5.1 ± 1.1 | 3.0-8.0 | 15.4 |
|  | Repeat 3 | 6.8±0.8 | 3.8±0.8 | 5.0 ± 1.1 | 3.0-8.0 | 14.7 |
| Elrose | 2014 | 7.5±0.5 | 4.0±0.0 | 5.3 ± 0.9 | 4.0-7.0 | 13.2 |
| Elrose | 2015 | 7.0±0.0 | 3.6±0.2 | 5.5 ± 1.0 | 3.0-8.5 | 18.4 |
| Saskatoon | 2017 | 8.0±0.0 | 4.5±0.3 | 6.3±1.3 | 3.0-9.0 | 20.3 |

Supplementary Table 1: Phenotypic variations of the CPR-02 population for reaction to ascochyta blight in the greenhouse (three repeats) and three years (2014-2015-2017) and two locations (Elrose and Saskatoon, SK).

Supplementary Table 2: Pearson correlation coefficients for ascochyta blight ratings under field conditions at Elrose, SK 2014 and 2015, and Saskatoon, SK 2017 as well as under greenhouse (GH) screenings.

|  | ELROSE 2014 | ELROSE 2015 | SASKATOON 2017 | GH |
| --- | --- | --- | --- | --- |
| ELROSE 2014 |  | 0.62*** | 0.45*** | 0.4*** |
| ELROSE 2015 |  |  | 0.47*** | 0.51*** |
| SASKATOON 2017 |  |  |  | 0.29** |
| GH |  |  |  |  |

** and *** indicate significant at P *<* 0.01 and 0.001, respectively.

Supplementary Table 3: Summary of genomic position and intervals of eight QTLs for ascochyta blight resistance in the chickpea genome.

| **QTL** | **Chromosome*** | **Genomic location of QTL flanking SNP markers^*^** | **QTL interval in bp** |
| --- | --- | --- | --- |
| qAB2.1 | Ca2 | 14258590-17335867 | 3077277 |
| qAB2.2 | Ca2 | 18250331-19241634 | 991303 |
| qAB2.3 | Ca2 | 20675628-20836067 | 160439 |
| qAB3.1 | Ca3 | 15444471-21346384 | 5901913 |
| qAB4.1 | Ca4 | 6022188-8584363 | 2562175 |
| qAB4.2 | Ca4 | 21540251-26669759 | 5129508 |
| qAB5.1 | Ca5 | 20906121-21049846 | 143725 |
| qAB6.1 | Ca6 | 43994721-54348174 | 10353453 |

^*^based on the CDC Frontier genome assembly V 2.6.3 (<http://www.cicer.info/cgi-bin/gb2/gbrowse/kabuliV2.6.3/>): Edwards (2016): Improved kabuli reference genome. CyVerse Data Commons. Dataset. <http://doi.org/10.7946/P2G596>

Supplementary Table 4: 100 bp flanking sequences of the SNP/InDels in the candidate genes.

| **KASP marker** | **100bp SNP/InDel flanking sequences (5’-3’)** |
| --- | --- |
| Ca2-GDSL2 | ACTCACTTGTAGATGTGGGAAACAACAATTACTTAACACTTTCAATTGTTAAGGCAAATCATCGTCACTATGGTATTGATTTTCTTAACCACAAACCAAC**[A/T]**GGAAGATTTAGCAACGGAAAGAATGCTGCTGATTTTATTGGTAAAGTTATTCAAATTTTTATAGACCCAACTCTTCAAATAAATATTTTTCATATATCCA |
| Ca2-ABAR | AGACAACAATAAAAAAAAATTGAAACAATCAAAACTCTGTTACAAATG[AAAAAT/A]AAAATAAATTAACACTAGCAAGTAGCAACCATTGTAGGAAAGCAG**[C/T]**AGTTATCATCATTCCAAATGAAAGAAGAAAAGATAGAAGATATACATCAACGTTAATTTTAACAATGCACTTCTCTCTAATCTTCCAAAAAAAAAAAAAA |
| Ca2-PEI | AGGGCGGTCTTCATTAACAATTATTTTGATGATTATATTCAACGAGATGGATATATGATATGGAAACCTAATGAAACAAACACTGAAAACTCGTACTTCG**[T/C]**TGAGTTTGGAAATATTGGACCTGGTGCCAATGCTACATCAAGAGTTAAATGGGCAAAAGGTGTCATTACCAAAGAGGAAGCTGTTAGATTTACTACTGAA |
| Ca4-ER2 | TCGAGTTAGCAATTTCAATGGTGACACGCCTCGTGAGGCGTGTTACGCAATACTTGTTTTGGTAATGCATAGTGGAGATGTGAGAATTTGGAGTAATCA**[G/A]**GAGTTGGAGATAGTTGAGGTGGTTGCTGATCAGGTAGCTGTTGCTCTTTCTCATGCTGCGGTTCTCGAGGAATCGCAGCTTATGAGAGAGAAATTAGAAG |
| Ca5-BTB | AGTTTTAGGGAGATTAATTTGAATTTCACCTGCAAAGAGGGACCTTGCAAGAATCTTGGTGAAGGCAAATGGAAGAGTGAAGTCTAAAGAGTTGCCACAT**[CCG/C]**CTTACAACGCAAGCACCCTCCCTTTAGATTCCTCTTACACGTGGCAAAGTGTCTTATCAAAACCTGCAAACCTTCGCACGTGGAGAATTTTGAACACG |

Supplementary Table 5: Genomic location of previously identified QTLs for ascochyta blight resistance in chickpea. QTL flanked microsatellites/ simple sequence repeats (SSR) marker sequences were used to locate QTLs position on the eight pseudomolecules (assembled chromosome) of CDC frontier reference genome assembly version 2.6.3. This information was used to compare QTLs identified in the present study with the earlier reposted QTLs in the different genetic background.

| **Chromosome*** | **QTL** | **QTL lined markers** | **Genomic**  **location (bp)*** | **References** |
| --- | --- | --- | --- | --- |
| Ca2 | QTL_AR3_ | TA37-GA16 | 4794488-20725707 | Cho et al. 2004 |
|  |  | TR13/TR19-TA110 | 13606125-35645662 | Anbessa et al. 2009 |
|  |  | Ein3 | 18970969 | Madrid et al. 2014 |
|  |  | Ca2v2.6p18250143_T/A  (NBS-LRR) | 18250143 | Deokar et al. 2018 |
| Ca3 | QTL_AR4_ | TS19-TA64 | 30120220-37876189 | Tar'an et.al.,2007 |
|  |  | TA64-TR26 | 37876000-38224538 | Anbessa et.al.,2009 |
| Ca4 | QTL_AR1_ | NCPGR91-GAA47 | 4411370-8006877 | Madrid et al. 2012 |
|  |  | GA24-GAA47 | 8006877-8802468 | Cho et al. 2004 |
|  |  | CaETR-1 (LOC101508648) | 4411499 | Madrid et al. 2012 |
|  |  | CaNIP18-CaNIP12, | 4314671-4506563 | Kumar et al 2018 |
|  |  | CaSTMS11-TA130 | 8802388-15658926 | Sabbavarapu et.al., 2013 |
|  |  | scaffold1758p1006151-scaffold405p3450200 | 6907594-13443128 | Daba et al. 2016 |
|  | QTL_AR2_ | TR20-TA72 | 22340178-43563684 | Udupa et.al., 2003 |
|  |  | TA146-TA72 | 24367557-43563684 | Iruela et.al.,2006 |
|  |  | TA2-TA146 | 24367557-41886687 | Tar'an et.al.,2007 |
|  |  | Ca4v2.6p26669292_T/G (ER2) | 26669292 | Deokar et al. 2018 |
| Ca6 | QTL_AR5_ | TA106-H1I16 | 685506-12381876 | Sabbavarapu et.al., 2013 |
|  |  | TA106-CaM0244 | 685506-2274651 | Sabbavarapu et.al., 2013 |
|  |  | TA22-TA80 | 5494172-54941700 | Tar'an et.al.,2007 |
| Ca8 | QTL_AR6_ | GA6-TS45 | 1428214-5196641 | Anbessa et.al.,2009 |

^*^ Pseudochromosomes numbers (Ca1 to Ca8) and positions in bp are based on the CDC Frontier reference genome assembly V 2.6.3 (<http://www.cicer.info/databases.php>)
